# Isokinetic Analysis of Fe_41_Co_7_Cr_15_Mo_14_Y_2_C_15_B_6_ Bulk Metallic Glass: Effect of Minor Copper Addition

**DOI:** 10.3390/ma13173704

**Published:** 2020-08-21

**Authors:** Parisa Rezaei-Shahreza, Amir Seifoddini, Saeed Hasani, Zahra Jaafari, Agata Śliwa, Marcin Nabiałek

**Affiliations:** 1 Department of Mining and Metallurgical Engineering, Yazd University, Yazd 89195-741, Iran; parisarezaei88@yahoo.com (P.R.-S.); seifoddini@yazd.ac.ir (A.S.); hasani@yazd.ac.ir (S.H.); z.jafari1991@yahoo.com (Z.J.); 2 Division of Materials Processing Technology and Computer Techniques in Materials Science, Silesian University of Technology, 44-100 Gliwice, Poland; Agata.Sliwa@polsl.pl; 3 Department of Physics, Faculty of Production Engineering and Materials Technology, Czestochowa University of Technology, 42-200 Częstochowa, Poland

**Keywords:** bulk metallic glasses (BMGs), crystallization kinetic, isokinetic analysis, nucleation and growth, JMAK method

## Abstract

In the present study, (Fe_41_Co_7_Cr_15_Mo_14_Y_2_C_15_B_6_)_100−x_Cu_x_ (x = 0, 0.25 and 0.5 at.%) amorphous alloys were prepared by copper-mold casting. To clarify the effect of the minor addition of copper on the mechanism of nucleation and growth during the crystallization process, an isokinetic analysis was performed. The activation energies (*E*) of the various crystallization stages were calculated by using theoretical models including Kissinger–Akahira–Sunose (KAS), Flynn–Wall–Ozawa (FWO), Augis–Bennett and Gao–Wang methods. In addition, Augis–Bennett, Gao–Wang and Matusita methods were used to investigate the nucleation and growth mechanisms and to determine other kinetic parameters including Avrami exponent (*n*), the rate constant (*K_p_*) and dimensionality of growth (*m*). The obtained results revealed that the activation energy—as well as thermal stability—was changed with minor addition of copper. In addition, the obtained Avrami exponent values were confirmed by Johnson–Mehl–Avrami–Kolmogorov (JMAK) method. The research findings demonstrated that the value of Avrami exponent is changed with minor addition of copper, so that the Avrami exponents of all crystallization stages, except the second peak for copper-free amorphous alloy, were equal to integer values ranging from two to four, indicating that the growth mechanisms were controlled by interface. Moreover, the kinetic parameters of *n* and *b* for all peaks were increased by an increase in crystallization temperature, which can be attributed to the increase in the nucleation rate.

## 1. Introduction

In recent years, many attempts have been made to generate new amorphous alloys and bulk metallic glasses (BMGs) with better properties and performance [1,2,3]. These efforts have led to the design and development of advanced BMGs with special properties such as high strength and hardness [4,5,6,7], relatively good corrosion resistance [8,9,10] and excellent magnetic properties [11,12,13]. Today, these materials play an important role in technological innovation because of wide range of their applications [14,15,16]. Meanwhile, Fe-based BMGs have attracted the tremendous attention of many researchers not only for their special properties, but also for their low cost [17,18,19,20,21,22].

Thermal stability and kinetic studies of crystallization process in the amorphous structures are known as the attractive and practical subjects [23,24], so that kinetic studies have a special and crucial role in determining the production parameters in order to produce an alloy with desirable structure and properties [25,26]. For instance, in BMGs with a maximum nucleation and the minimum growth rates, crystallization process can take place partially by controlling the kinetic parameters and as a result an amorphous matrix nanocomposite can be produced with excellent mechanical and magnetic properties [27,28,29]. On the other hand, the presence of alloying elements can strongly control the size of crystalline particles during annealing process [30,31,32,33,34,35,36,37]. For instance, Lesz et al. [32] studied the effect of Ni addition on the thermal properties of a Fe-based amorphous alloy. They showed that the activation energy of crystallization process was increased from 564 to 623 kJ/mol with the addition of this alloying element; indication that an increase in the glass-forming ability (GFA).

Recently, Fe_41_Co_7_Cr_15_Mo_14_Y_2_C_15_B_6_ (at.%) BMG has been introduced with a high GFA (super-cooled liquid region; (Δ*T_x_* = 94 K)), high hardness (1368.4 H_V_), and good strength (2217 MPa). In addition, the minor addition of copper improved the properties of this BMG due to the change of its thermal stability [7,17,37]. Although, the triple kinetic parameters of the crystallization process including the activation energy (*E*), pre-exponential factor (*A*) and reaction model (*f*(*α*)) were determined [38], no comprehensive investigation has been done into the isokinetic analysis of crystallization process of this BMG to determine more kinetic parameters and, therefore, there exists a knowledge gap. In the present study, an isokinetic analysis is done to determine the effect of presence of copper on the isokinetic parameters including Avrami exponent (*n*) and dimensionality of growth (*m*) for partial crystallization process by using thermal analysis techniques. For this purpose, other kinetic methods such as isoconversional Augis–Bennet [39], Gao–Wang [40], Kissinger–Akahira–Sunose (KAS) [41] and Flynn–Wall–Ozawa (FWO) [42,43] methods and isokinetic Johnson–Mehl–Avrami–Kolmogorov (JMAK) method [44,45,46] are used.

## 2. Materials and Methods

Multicomponent alloys with nominal compositions of (Fe_41_Co_7_Cr_15_Mo_14_Y_2_C_15_B_6_)_100−x_Cu_x_ (x = 0, 0.25 and 0.5 at.%) were synthesized by using vacuum-arc melting under a controlled argon atmosphere by using the high-purity raw materials (≥99.999%). Then, the master alloy ingots were reverse-remelted at least four times to ensure the reproducibility in the results. Cylindrical samples with a diameter of 2 mm and a length of 70 mm were produced by suction copper-mold casting. Solid-state processes have been extensively studied by using thermal analysis techniques [47,48,49]. Therefore, the thermal stability and isokinetic analysis of the as-cast specimens were evaluated by using a differential scanning calorimetry (DSC, NETZSCH DSC 404C, NETZSCH-Gerätebau GmbH, Selb, Germany) at continuous heating rates of 5, 10 and 20 °C/min. In addition, the as-cast specimens were heated in nonisothermal condition by DSC at a heating rate of 20 °C/min up to the maximum temperature of each peak simultaneously with argon flow. Phase analysis of the as-cast and annealed specimens was identified by X-ray diffraction method using a X’Pert MPD Philips diffractometer with Co-kα radiation. Moreover, to validate the kinetic results, a microstructural observation and the crystallites size distribution was performed by using a field emission scanning electron microscope (FE-SEM, MIRA 3, TESCAN, Czech Republic) at an accelerate voltage of 15 kV and an optical microscopy (OM, Olympus BX60M, Tokyo, Japan). To determine the crystallites size distribution, at least three FE-SEM images from different positions of every specimen were randomly selected in order to obtain a reliable distribution of the particle size. For this purpose, the microstructural image processing software (MIP 4 student; Nahamin Pardazan Asia, Iran) was used.

## 3. Results

**Figure 1:**
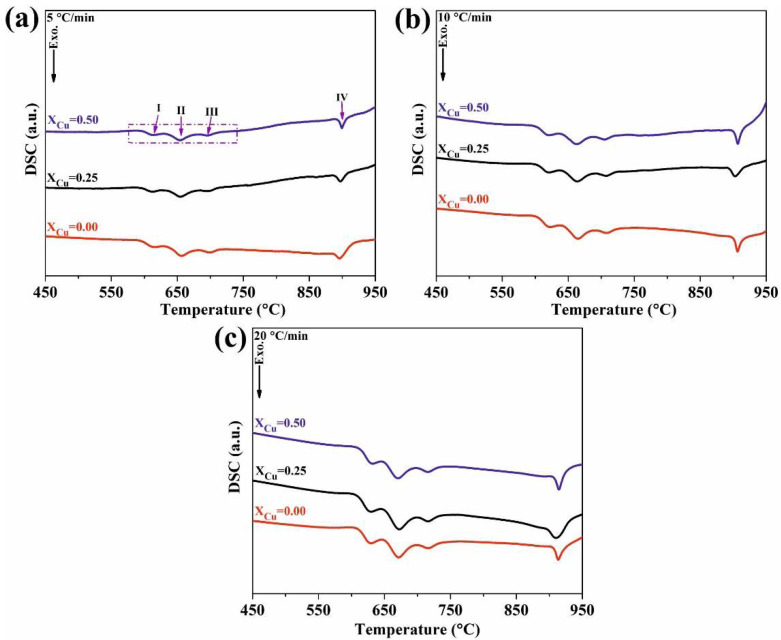
Differential scanning calorimetry (DSC) curves of the investigated bulk metallic glasses (BMGs) at heating rates of (**a**) 5 °C/min; (**b**) 10 °C/min; (**c**) 20 °C/min.

Figure 1 presents DSC curves of the investigated BMGs. As seen, there were four exothermic peaks for each BMG and at every heating rate. In addition, it has been shown that with an increase in heating rate, the critical temperatures such as glass transition temperature (*T_g_*), onset crystallization temperature (*T_x_*) and crystallization peak temperature (*T_p_*) shift to high temperatures, which is in good agreement with the results obtained by the researchers [50,51,52,53]. The characteristic temperatures are listed in Table 1. As seen, all characteristic temperatures shifts to higher temperatures; indicating that the crystallization process depends on the heating rate caused by the fact that crystallization is a thermally activated process. In other words, the crystallization temperature of amorphous alloy exhibit strong dependence on the heating rate, which can be attributed to thermally activated process [54,55,56].

**Figure 2:**
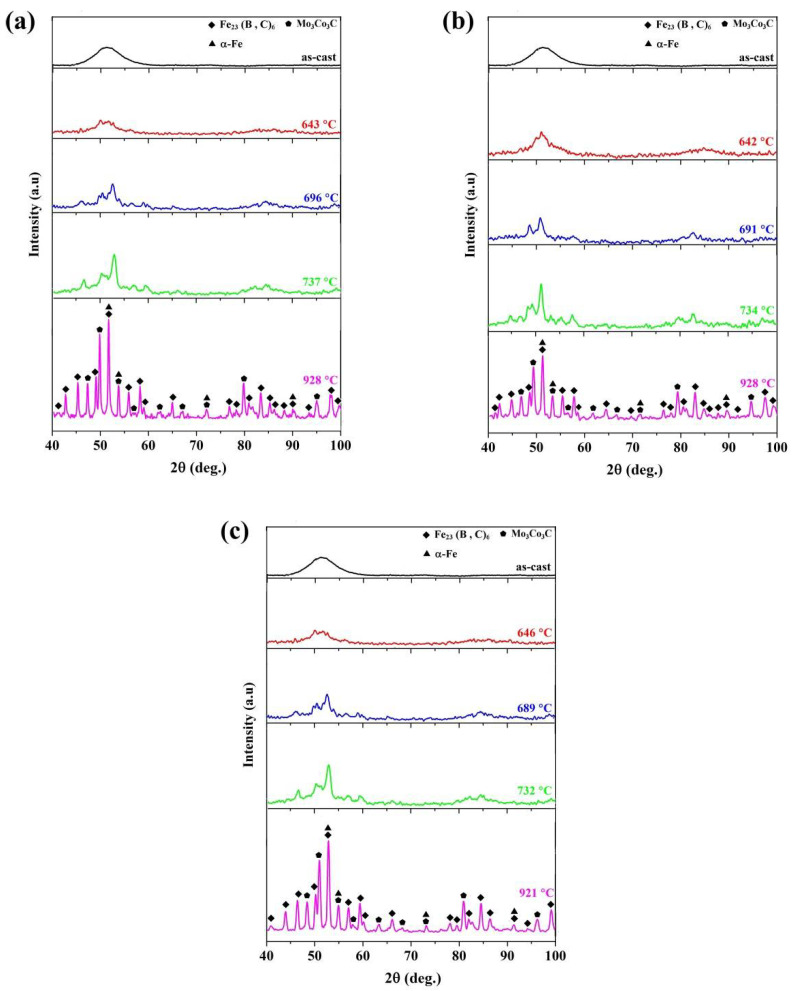
XRD patterns of the as-cast (Fe_41_Co_7_Cr_15_Mo_14_Y_2_C_15_B_6_)_100−x_Cu_x_ (x = 0, 0.25 and 0.5 at.%) BMGs and the annealed specimens up to the maximum temperature of each peak. (**a**) x = 0 at.%; (**b**) x = 0.25 at.%; (**c**) x = 0.5 at.%.

On the other hand, as listed in Table 1, it can be seen that *T_g_* shifts to higher temperatures by minor addition of copper. As previously discussed in detail, the thermal stability and glass forming ability (GFA) is increased by the minor addition of copper [17], which is in good agreement with that of the obtained for *T_g_*. Figure 2 depicts the X-ray diffraction patterns of the as-cast and the annealed samples. As seen, the patterns of the all three as-cast BMGs exhibit a typical broad hump at 2*θ* = 50°, demonstrating a fully amorphous microstructure. However, the XRD patterns of the annealed specimens consist of sharp Bragg peaks, which confirm the formation of crystalline precipitates during the crystallization process. As seen in Figure 2, an increase in crystallization temperature leads to the crystalline phases including α-Fe, Fe_23_(B, C)_6_, and Mo_3_Co_3_C formed in this alloy up to ∼950 °C, which confirms that the peaks of DSC curves are related to the crystallization process.

### 3.1. Isoconversional Methods

The isoconversional methods are often used to describe their kinetic parameters, evaluate the results of thermal analysis data and provide more insight into the complex reaction mechanism [57,58]. The calculation of activation energy without having to determine the reaction model is one of the advantages of these methods. The activation energy is used to describe the required energy of thermal activation leading to atomic movement [59,60]. As know, the activation energy can be determined in two different ways of local and apparent by using these methods.

#### 3.1.1. Local Activation Energy

Local activation energy (*E_α_*) describes the dependence of the activation energy on degree of conversion (*α*). Therefore, to estimate the activation energy at any degree of conversion is very important for the crystallization proceeding. This can clarify the nucleation and growth activation energies required for nonisothermal crystallization. In other words, the local activation energy can be used to determine whether the reaction is single-step or multistep. In other words, changes of activation energy for the reactions controlled by nucleation and growth mechanism (Avrami model) indicate that nucleation and growth mechanisms are changing as the reaction progresses [61,62] and this reaction is a single step reaction. Therefore, it is possible to provide a degree of complexity of the transformation mechanism of the dependence of *E_α_* on *α*. Hence, to investigate the effect of copper addition on the nucleation and growth mechanisms of Fe_41_Co_7_Cr_15_Mo_14_Y_2_C_15_B_6_BMG, an isokinetic analysis is performed. For this purpose, according to the data extracted from the DSC curves [17,49], the degree of conversion versus temperature (*T*) can be obtained for the various crystallization stages, and then the dependence of *E_α_* on a wide range of *α* is calculated by using FWO [42,43] and KAS [41] isoconversional methods. The KAS and FWO are derived from integral isoconversional methods based on Equations (1) and (2), respectively:

(1) $$ln(\frac{\beta }{T^{2}})=\mathrm{constant}-\frac{E_{\alpha }}{RT}$$

(2) $$\ln\beta =\mathrm{constant}-1.0516\frac{E_{\alpha }}{RT}$$

where *E_α_* (kJ/mol) is the local activation energy; *R* (J/mol·K) is the universal gas constant; *β* (°C/min) is the heating rate; and *T* (K) is the absolute temperature. According to Equations (1) and (2), the *E_α_* values are calculated from the slopes of ln(*β*/*T*^2^) and ln(*β*) versus 1000/*T*, respectively.

**Figure 3:**
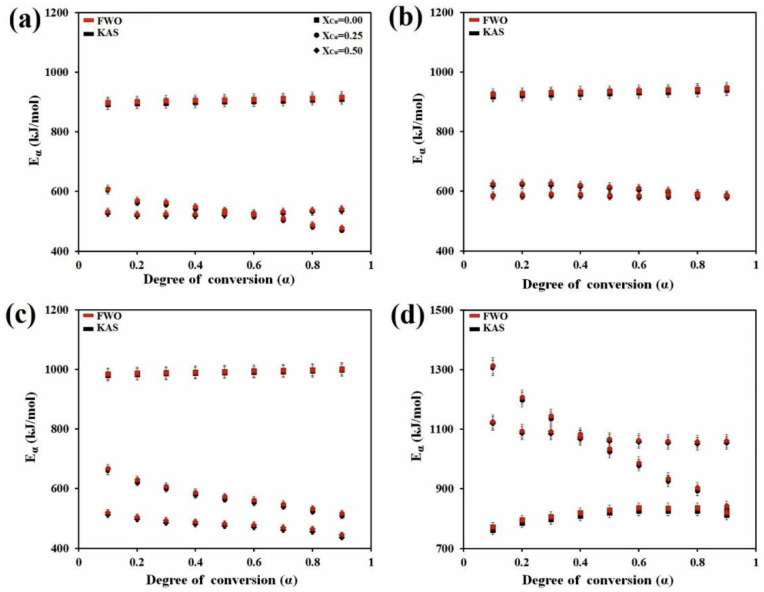
Dependence of *E_α_* on *α* examined for (Fe_41_Co_7_Cr_15_Mo_14_Y_2_C_15_B_6_)_100−x_Cu_x_ (x = 0, 0.25 and 0.5 at.%) by using Kissinger–Akahira–Sunose (KAS) and Flynn–Wall–Ozawa (FWO) methods for peak (**a**) I, (**b**) II, (**c**) III and (**d**) IV.

Figure 3 displays the dependence of *E_α_* vs. *α* for all crystallization stages of all three (Fe_41_Co_7_Cr_15_Mo_14_Y_2_C_15_B_6_)_100−x_Cu_x_ (x = 0, 0.25 and 0.5 at.%) alloys, which were obtained by using of the KAS and FWO methods.

As can be seen, the local activation energies for all crystallization stages of copper-free specimen and specimen containing 0.5 at.% copper was found to be practically independent on *α* in a very wide conversion range, which means that these processes are one-step reactions, while the results obtained for specimen containing 0.25 at.% copper show that the activation energies of the first, third, and fourth stages of crystallization process change with the extent of conversion. The dependence of *E_α_* on *α* suggests that crystallization stages in this specimen undergo as multistep kinetics reactions. Moreover, the average values of activation energies are presented in Table 2. As presented, the results obtained by these two methods are in good agreement with each other. It is accepted that addition of alloying elements (i.e., copper) can change the kinetic parameters of reaction such as activation energy due to the formation of short range ordering (SRO) regions [63,64]. For instance, the average of the local activation energy for the fourth stage of crystallization of the (Fe_41_Co_7_Cr_15_Mo_14_Y_2_C_15_B_6_)_100−x_Cu_x_ (where x = 0.5 at.%) is the highest activation energy value compared to the fourth stages of the other two alloys. It is notable that the higher energy barrier, the slower reaction [65,66,67,68,69]. On the other hand, the activation energies for the crystallization process of the investigated BMGs are more than the other Fe-based BMGs [70,71,72,73].

#### 3.1.2. Apparent Activation Energy

The activation energy at peak temperature (*T_p_*) of each crystallization step is called apparent activation energy (*E_p_*). This kinetic parameter represents the value of activation energy when the reaction is the fastest [74,75]. To calculate the apparent activation energy of investigated alloys, Augis–Bennet [39] (Equation (3)) and Gao–Wang [40] (Equation (4)) methods were used, which were developed based on Kissinger and Friedman methods, respectively:

(3) $$\ln\left(\frac{\beta }{T_{p}}\right)=-\frac{E_{p}}{RT_{p}}+\ln A$$

(4) $$ln{\left(\frac{d\alpha }{dt}\right)}_{p}=-\frac{E_{p}}{RT_{p}}+const$$

where *E_p_* (kJ/mol) is the apparent activation energy at the peak temperature; *T*_0_ (K) is the onset crystallization temperature; (*dα*/*dt*)*_p_* is the maximum crystallization rate at *T_p_*.

**Figure 4:**
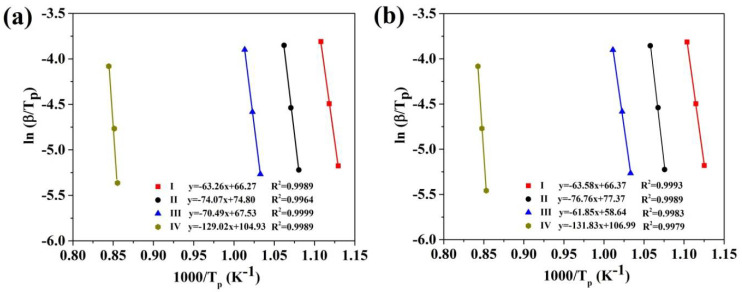
Plots of ln(*β/T_p_*) vs. 1000/*T_p_* for all crystallization stages of (Fe_41_Co_7_Cr_15_Mo_14_Y_2_C_15_B_6_)_100−x_Cu_x_ amorphous alloys; (**a**) x = 0.25 at.%; (**b**) x = 0.5 at.%.

**Figure 5:**
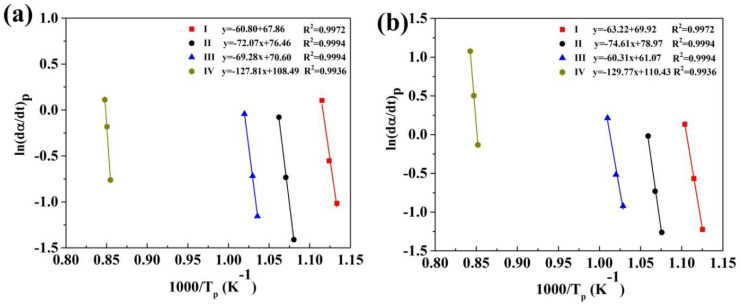
Plots of ln(*dα*/*dt*)*_p_* vs. 1000/*T_p_* for all crystallization stages of (Fe_41_Co_7_Cr_15_Mo_14_Y_2_C_15_B_6_)_100−x_Cu_x_ amorphous alloys; where x= (**a**) 0.25 at.%; (**b**) 0.5 at.%.

Based on these equations, the value of *E_p_* is evaluated from the slops of a plots of ln(*β/T_p_*) and ln(*dα/dt*)*_p_* vs. 1000/*T_p_*, respectively. For instance, the curves of ln(*β*/*T_p_*) vs. 1000*/T_p_* curves for all four crystallization stages of (Fe_41_Co_7_Cr_15_Mo_14_Y_2_C_15_B_6_)_100−x_Cu_x_(x = 0.25 at.% and 0.5 at.%) are presented in Figure 4. In addition, Figure 5 indicates the curves of ln(*dα/dt*)*_p_* vs. 1000/*T_p_* for (Fe_41_Co_7_Cr_15_Mo_14_Y_2_C_15_B_6_)_100−x_Cu_x_ (where x = 0.25 at.% and 0.5 at.%) BMGs.

The values of *E_p_* obtained by these methods are listed in Table 2. As can be seen, the average of local activation energy for every single-step stage is in a good agreement with its apparent activation energy, while the apparent activation energy values are significantly different from the local activation energy in the multistep stage.

### 3.2. Nucleation and Growth Mechanisms

Generally, crystallization processes are controlled by nucleation and growth phenomena [76,77,78]. Therefore, understanding the mechanisms of nucleation and growth during the crystallization process is essential to control the microstructure and its dependent mechanical and magnetic properties [61,79]. Among the kinetic parameters related to the nucleation and growth mechanisms, the calculation of *n*, *m*, and *K_p_* can be necessary to do a comprehensive kinetic analysis.

#### 3.2.1. Avrami Exponent and the Rate Constant

Augis–Bennet [39] and Gao–Wang [40] methods are commonly used to obtain the kinetic parameters including *n* and *K_p_*. The *n* value can be calculated by using Equation (5), which was developed by Augis & Bennett method.

(5) $$n=2.5\frac{T_{p}^{2}}{\Delta T\left(\frac{E_{p}}{R}\right)}$$

where Δ*T* is the full width of the exothermic peak at the half maximum intensity of crystallization peak. Moreover, the kinetic parameters such as *K_p_* and *n* can be obtained by using the Gao–Wang method by using Equations (6) and (7), respectively.

(6) $$K_{p}=\frac{\beta E_{p}}{RT_{p}^{2}}$$

(7) $${\left(\frac{d\alpha }{dt}\right)}_{p}=0.37nK_{p}$$

As seen, to calculate the *n* values by using Gao–Wang method (Equation (7)), the *K_p_* parameter should be calculated (Equation (6)).

The Avrami exponent and *K_p_* for every crystallization stage of the investigated BMGs is listed in Table 2. Considering the *n* values calculated by these two methods, it can be concluded that the values of Avrami exponent change by minor addition of copper.

Moreover, to verify accuracy of the obtained results, the isokinetic JMAK method was used [44,45,46], which can be expressed as:

(8) $$n(\alpha )=\frac{R\partial ln(-ln(1-\alpha ))}{E_{\alpha }\partial (\frac{1}{T_{\alpha }})}$$

**Figure 6:**
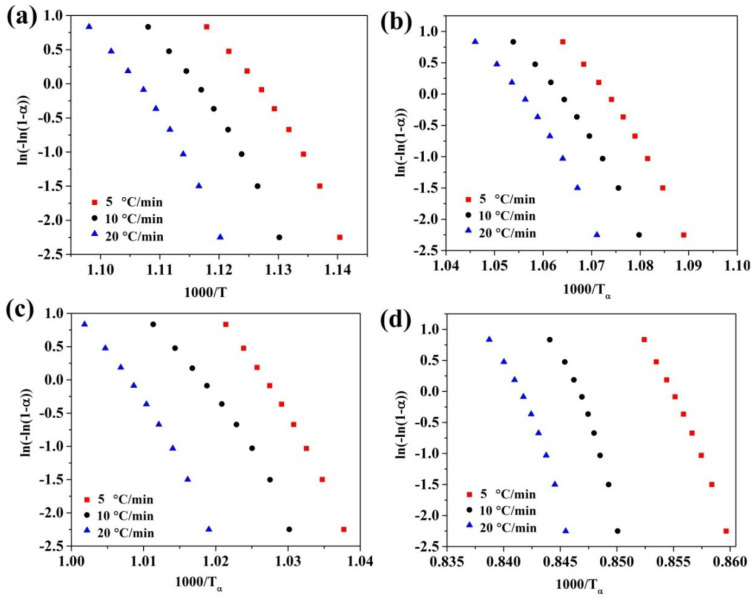
Plots of ln(−ln(1−*α*)) vs. 1000/*T_α_* for (Fe_41_Co_7_Cr_15_Mo_14_Y_2_C_15_B_6_)_100–0.25_Cu_0.25_BMGs with various heating rates. (**a**) Peak I; (**b**) peak II; (**c**) peak III; (**d**) peak IV.

**Figure 7:**
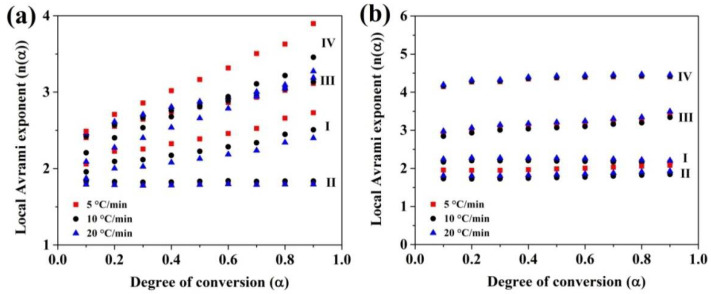
Local Avrami exponent (*n*(*α*)) as a function of *α* for all crystallization stages of (Fe_41_Co_7_Cr_15_Mo_14_Y_2_C_15_B_6_)_100−x_Cu_x_ amorphous alloys; where (**a**) 0.25 at.%; (**b**) 0.5 at.%.

In order to obtain the local Avrami exponent (*n*(*α*)) under nonisothermal crystallization kinetic analysis by using JMAK method, the plots of ln(−ln(1−*α*)) vs. 1000/*T_α_* are needed and then the *n*(*α*) can be obtained by using Equation (8). For instance, the plots of ln(−ln(1−*α*)) vs. 1000/*T_α_* for all four crystallization stages of (Fe_41_Co_7_Cr_15_Mo_14_Y_2_C_15_B_6_)_100−0.25_Cu_0.25_ amorphous alloy in different heating rates are indicated in Figure 6. In addition, Figure 7 shows the plots of *n*(*α*) vs. *α* for all four crystallization stages of (Fe_41_Co_7_Cr_15_Mo_14_Y_2_C_15_B_6_)_100−0.25_Cu_0.25_ amorphous alloy in different heating rates.

**Figure 8:**
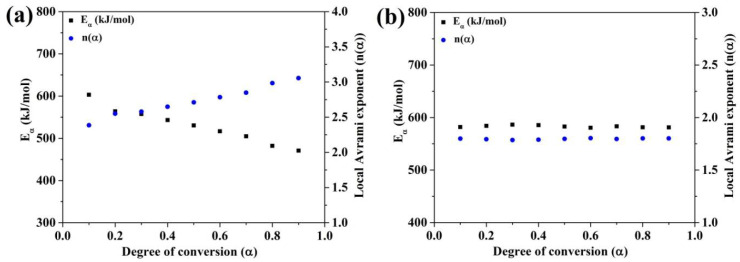
Local Avrami exponent (*n*(*α*)) and *E_α_* as a function of *α* for (**a**) the first and (**b**) second crystallization stages of (Fe_41_Co_7_Cr_15_Mo_14_Y_2_C_15_B_6_)_100−0.25_Cu_0.25_ BMG.

As shown in Figure 7, *n*(*α*) is constant over a wide range of *α* at the second peak of (Fe_41_Co_7_Cr_15_Mo_14_Y_2_C_15_B_6_)_100−0.25_Cu_0.25_ BMG and the all four crystallization stages of (Fe_41_Co_7_Cr_15_Mo_14_Y_2_C_15_B_6_)_100−0.5_Cu_0.5_ BMG. It is accepted that variation of *n*(*α*) vs. *α* shows a multistep reaction, while this kinetic parameter is constant for single-step reaction. Figure 8 displays the plots of *E_α_* and *n*(*α*) vs. *α* for the first and second crystallization stages of the (Fe_41_Co_7_Cr_15_Mo_14_Y_2_C_15_B_6_)_100−0.25_Cu_0.25_ amorphous alloy.

As shown in Figure 8a, the *n*(*α*) and *E_α_* change as the reaction progresses, indicating that this reaction is a multistep reaction, while as shown in Figure 8b, the *E_α_* and *n*(*α*) for the second crystallization stage are constant in a wide range of *α*, indicating that this reaction is a single-step reaction. The average values of *n*(*α*) for all three heating rates are listed in Table 2.

In the crystallization process, the activation energy is related to overcome the potential barrier for nucleation and growth, which can determine the rate of crystallization process [80,81,82]. Therefore, with a decrease in the activation energy, the number of nucleation sites increases and then the diffusion process becomes easier [83]. Therefore, it can lead to more progress in crystallization and increases Avrami exponent.

#### 3.2.2. Relationship between *n* & *m* Parameters

The growth dimension (*m*) is calculated using the Matusita equation [79,80] based on the following equation:

(9) $$\ln(\beta )=-1.052\frac{m}{n}\frac{E}{RT}-\frac{1}{n}\ln(-\ln(1-\alpha ))+\mathrm{const}.$$

where, the ratio of *m/n* can be obtained by plotting the ln(*β*) vs. 1000/*T*. For this purpose, the activation energy obtained by the Gao–Wang method is used in Equation (9). The *n*(*α*) value of JMAK method was used to determine the growth dimension (*m*). The values of *m* for every crystallization stage of the investigated BMGs are presented in Table 3.

In addition, the Ranganathan–Heimendahl equation [81,82] can be used to investigate the relationship between nucleation and growth mechanism, which is presented as followed:

(10) n=pm+b

where *b* is a nucleation index, which *b* = 0 and *b* = 1 indicate the nucleation rate will be zero and constant, respectively. However, the nucleation rate will be decreasing and increasing for 0 < *b* < 1 and *b* > 1, respectively. The *p* parameter referred to the growth index and the value of this parameter can be considered as 0.5 and 1 for diffusion and interfaced controlled growth, respectively [83,84].

The obtained values of *b* and *p* for the all crystallization stages of the investigated BMGs are listed in Table 3. As presented, the value of Avrami exponent changes with minor addition of copper, so that the Avrami exponents of all crystallization stages except the second peak for copper-free BMG are equal to integer values ranging from 2 to 4. As seen, the value of *p* parameter is equal to 1 for all four crystallization stages of the investigated BMGs. It means that these stages have an interface-controlled growth mechanism. In addition, the value of nucleation index related to the first, second and third crystallization stages for all investigated BMGs are equal to 0, while for the fourth crystallization peaks of three BMGs are equal 1, which indicating that the nucleation rate is increased in this stage.

### 3.3. Microstructural Observations

**Figure 9:**
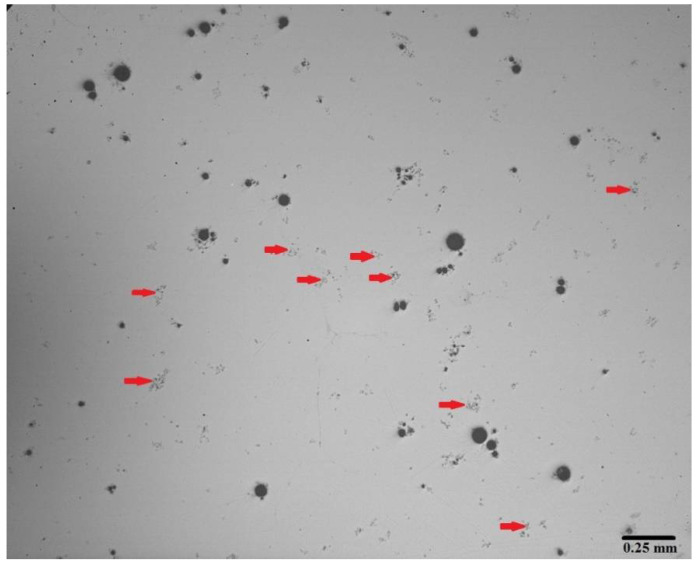
Micrograph related to the pre-existing clusters in the as-cast copper-free BMG.

**Figure 10:**
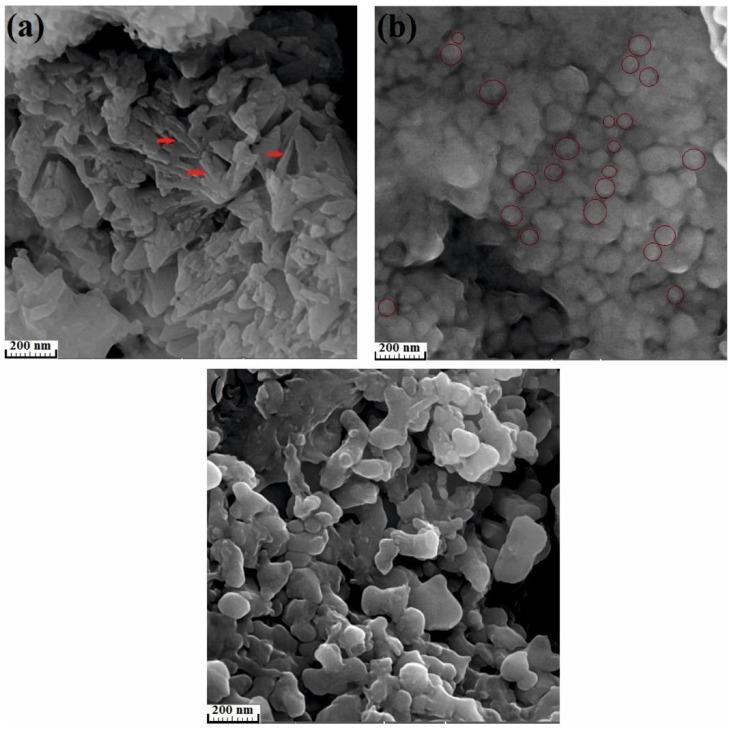
FE-SEM micrographs of the nanocrystalline phases formed in the amorphous matrix of (Fe_41_Co_7_Cr_15_Mo_14_Y_2_C_15_B_6_)_100−x_Cu_x_. (**a**) x = 0 at.% annealed up to temperature range of the first crystallization stage; (**b**) x = 0.25 at.% annealed up to temperature range of the third crystallization stage; (**c**) x = 0.5 at.% annealed up to temperature range of the fourth crystallization stage.

As listed in Table 3, the nucleation rate (*b*) for the first, second and third crystallization stages are calculated equal to 0 due to the presence of pre-existing clusters. For instance, these pre-existing clusters in the as-cast copper-free BMG are presented in Figure 9. The clusters exist in this sample are shown with red arrows. Also, Figure 10 displays FE-SEM micrographs of the nanocrystalline phases formed in the amorphous matrix of (Fe_41_Co_7_Cr_15_Mo_14_Y_2_C_15_B_6_)_100−x_Cu_x_(x = 0, 0.25 and 0.5 at.%) alloys annealed at temperature ranges of the first, third and fourth crystallization stages, respectively. As seen in Figure 1a, the plate precipitates are formed in the copper-free amorphous alloy by annealing up to temperature range of the first crystallization peak, which indicates a two-dimensional growth (*m* = 2). Therefore, microstructural observations confirm the accuracy of the results obtained by kinetic analysis (the Matusita equation). In addition, in Figure 10b,c, the formed spherical precipitates are shown in the BMGs of containing 0.25 at.% and 0.5 at.% copper annealed at the temperature ranges of the third and fourth crystallization peaks, respectively. As listed in Table 3, the growth dimensions for these samples are calculated equal to 3. Therefore, the formation of spherical crystalline precipitates in these samples is expected.

**Figure 11:**
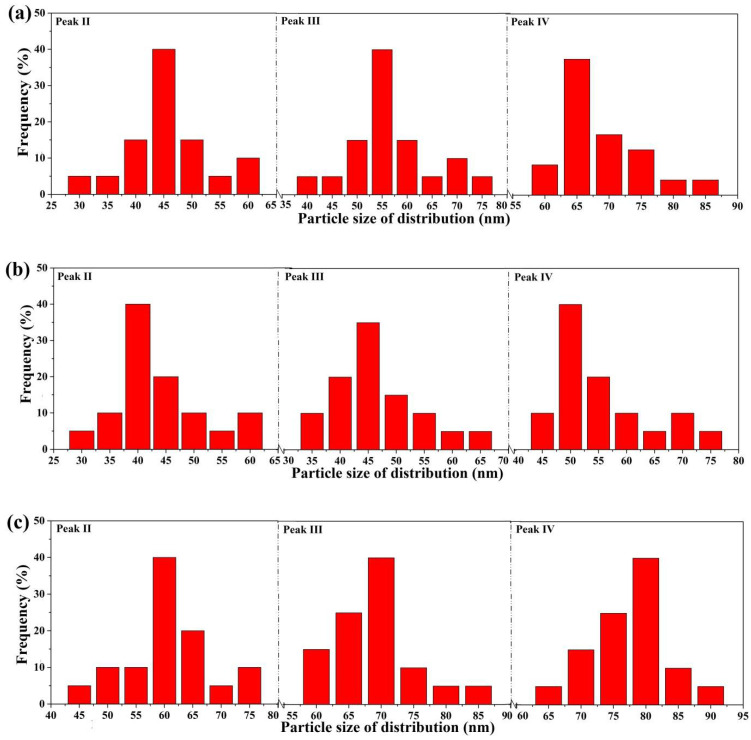
Size distribution of nanocrystallites formed in the specimens of (Fe_41_Co_7_Cr_15_Mo_14_Y_2_C_15_B_6_)_100−x_Cu_x_ BMGs. (**a**) x = 0 at.%; (**b**) x = 0.25 at.%; (**c**) x = 0.5 at.% annealed up to the maximum temperature of the second (II), third (III) and fourth (IV) peaks of crystallization, respectively.

Moreover, Figure 11 illustrates the size distribution of the formed nanocrystallites in the annealed specimens during the partial annealing. According to this figure, it is confirmed that the average size of nanocrystallites is increased with an increase in the annealing temperature. In other words, the size of nanocrystallites depends on the annealing temperature. On the other hand, it is shown that the size of nanocrystals in the same annealing temperature decreases in the presence of 0.25 at.% Cu compared with the Cu-free specimens and the alloy containing 0.5 at.% Cu. This results indicates that the optimal size of nanocrystallites can be formed in the presence of 0.25 at.% Cu, which can be due to the effect of Cu presence on the mechanism of nucleation and growth of crystalline phases. This phenomenon can improve its mechanical properties, which are discussed in detail elsewhere [7].

## 4. Conclusions

In this study, the effect of copper presence on the mechanisms of nucleation and growth for Fe_41_Co_7_Cr_15_Mo_14_Y_2_C_15_B_6_BMG was investigated by using various isoconversional and isokinetic methods. Activation energies of the investigated BMGs in various crystallization stages were measured by various kinetic methods such as; FWO, KAS, Augis–Bennett and Gao–Wang methods. Activation energies for three BMGs were obtained in the range of about 470 to 1100 kJ/mol. In addition, the kinetic parameter including *n*, *K_p_* and *m* were determined by using Augis–Bennett, Gao–Wang, Matusita and JMAK methods. The results revealed that the value of Avrami exponent is changed with minor addition of copper, so that the Avrami exponents of all crystallization stages except the second peak for copper-free amorphous alloy were equal to integer values ranging from two to four. Furthermore, the value of *p* parameter is equal to one for all four crystallization stages of three BMGs. Hence, it is confirmed that all peak of crystallization were controlled by the interface. In addition, the results showed that the *n* and *b* values of investigated BMGs for the fourth peaks of crystallization increased, indicating the nucleation rate is increased in this stage. Microstructural study confirmed the calculated kinetic results, so that plate and spherical crystalline precipitates was observed for the samples with two- and three-dimensional growth (*m* = 2 and 3), respectively.

## Figures and Tables

**Table 1:** Effect of both copper minor addition and heating rates on the characteristics temperatures of the investigated BMGs, extracted from DSC curves.

**X (at.%)**	**Heating Rate (°C/min)**	**Tg (°C)**	**Tx (°C)**	**Tm (°C)**	**Tl (°C)**	**Reference**
0.00	5	472	595	1105	1156	This work
	10	499	601	1108	1159	This work
	20	516	610	1112	1165	[17]
0.25	5	507	597	1108	1153	This work
	10	530	604	1111	1157	This work
	20	545	609	1114	1162	[17]
0.50	5	512	601	1107	1154	This work
	10	533	605	1109	1159	This work
	20	552	612	1113	1163	[17]

**Table 2:** Values of kinetic parameters including; activation energy (*E*), Avrami exponent (*n*) and the value of constant rate of maximum peak (*K_p_*) by using different methods for all crystallization peaks.

**Peak Number**	**X** **(at.%)**	***E*** ** (kJ/mol)**				**Heating Rate (°C/min)**	***K_p_***	***n***			**Ref** **erence**
		FWO	KAS	Augis and Bennet	Gao–Wang		Gao–Wang	Gao–Wang	JMAK	Augis and Bennet	
I	0.00	546.0 ± 11.0	559.3 ± 11.5	578.3 ± 2.4	583.2 ± 2.4	5	0.449	1.66	1.88 ± 0.03	1.71	[37]
						10	0.882	1.67	1.92 ± 0.03	1.82	[37]
						20	1.735	1.72	1.95 ± 0.04	1.90	[37]
	0.25	–	–	525.9 ± 3.7	505.5 ± 4.3	5	0.451	1.98	–	2.09	This work
						10	0.768	2.02	–	2.14	This work
						20	1.512	2.17	–	2.23	This work
	0.50	512.5 ± 3.4	±524.1 ± 3.5	528.6 ± 5.2	525.6 ± 7.2	5	0.413	1.99	2.18 ± 0.02	1.88	This work
						10	0.782	1.95	2.21 ± 0.02	1.97	This work
						20	1.540	2.00	2.25 ± 0.03	2.12	This work
II	0.00	616.2 ± 4.5	632.3 ± 4.7	627.6 ± 3.3	636.6 ± 3.3	5	0.445	1.43	1.48 ± 0.08	1.45	[37]
						10	0.875	1.45	1.49 ± 0.06	1.47	[37]
						20	1.724	1.49	1.53 ± 0.08	1.49	[37]
	0.25	578.0 ± 3.4	582.0 ± 3.7	615.8 ± 4.2	599.2 ± 5.1	5	0.355	1.82	1.79 ± 0.05	1.78	This work
						10	0.712	1.84	1.80 ± 0.07	1.82	This work
						20	1.354	1.86	1.83 ± 0.06	1.89	This work
	0.50	601.6 ± 3.5	605.9 ± 3.5	638.2 ± 8.3	620.3 ± 3.8	5	0.432	1.77	1.82 ± 0.08	1.74	This work
						10	0.723	1.83	1.95 ± 0.07	1.79	This work
						20	1.421	1.88	2.01 ± 0.08	1.82	This work
III	0.00	513.5 ± 2.3	591.6 ± 2.4	588.7 ± 5.1	592.7 ± 5.1	5	0.378	1.98	1.88 ± 0.13	1.78	[37]
						10	0.745	2.10	1.92 ± 0.12	1.82	[37]
						20	1.460	2.30	1.90 ± 0.11	1.89	[37]
	0.25	–	–	586.0 ± 7.2	575.9 ± 6.7	5	0.295	2.71	–	2.84	This work
						10	0.462	2.83	–	2.88	This work
						20	0.955	3.21	–	3.12	This work
	0.50	467.4 ± 4.6	474.8 ± 4.6	514.2 ± 6.5	501.4 ± 5.1	5	0.319	2.91	3.08 ± 0.12	2.79	This work
						10	0.55	2.93	3.16 ± 0.12	2.87	This work
						20	1.492	3.37	3.22 ± 0.13	3.12	This work
IV	0.00	826.5 ± 9.5	808.0 ± 9.5	929.2 ± 6.3	935.2 ± 6.3	5	0.412	3.31	3.71 ± 0.07	3.61	[37]
						10	0.810	3.85	3.85 ± 0.07	3.74	[37]
						20	1.600	4.20	3.91 ± 0.06	3.82	[37]
	0.25	–	–	1072.7 ± 8.2	1062.6 ± 7.3	5	0.565	2.83	–	2.97	This work
						10	0.954	2.93	–	3.07	This work
						20	1.566	4.09	–	3.12	This work
	0.50	1063.9 ± 5.7	1068.7 ± 3.4	1096.0 ± 3.8	1078.9 ± 6.9	5	0.552	3.98	4.29 ± 0.06	3.85	This work
						10	1.091	4.01	4.35 ± 0.08	4.12	This work
						20	1.576	4.22	4.39 ± 0.08	4.15	This work

**Table 3:** Nonisothermal crystallization kinetics data for nucleation and growth mechanism.

Peak Number	X (at.%)	Avrami Exponent (n)	Dimensionality of Growth (m)	Growth Index (p)	Nucleation Index (b)	Reference
I	0.00	2	2	1	0	[37]
	0.25	2	2	1	0	This work
	0.50	2	2	1	0	This work
II	0.00	1.5	1	1	0	[37]
	0.25	2	2	1	0	This work
	0.50	2	2	1	0	This work
III	0.00	2	2	1	0	[37]
	0.25	3	3	1	0	This work
	0.50	3	3	1	0	This work
IV	0.00	4	3	1	1	[37]
	0.25	3	2	1	1	This work
	0.50	4	3	1	1	This work
